# Phase II Trial Assessing the Repeatability and Tumor Uptake of [^68^Ga]Ga-HER2 Single-Domain Antibody PET/CT in Patients with Breast Carcinoma

**DOI:** 10.2967/jnumed.123.266254

**Published:** 2024-02

**Authors:** Odrade Gondry, Vicky Caveliers, Catarina Xavier, Laurens Raes, Marian Vanhoeij, Guy Verfaillie, Christel Fontaine, Katrien Glorieus, Jacques De Grève, Sofie Joris, Ine Luyten, Karen Zwaenepoel, Frederik Vandenbroucke, Wim Waelput, Sheeno Thyparambil, Ilse Vaneycken, Julie Cousaert, Sophie Bourgeois, Nick Devoogdt, Lode Goethals, Hendrik Everaert, Frank De Geeter, Tony Lahoutte, Marleen Keyaerts

**Affiliations:** 1 Department of Medical Imaging, Vrije Universiteit Brussel, Brussels, Belgium;; 2 Department of Nuclear Medicine, Universitair Ziekenhuis Brussel, Brussels, Belgium;; 3 Department of Surgical Oncology, Universitair Ziekenhuis Brussel, Brussels, Belgium;; 4 Department of Medical Oncology, Universitair Ziekenhuis Brussel, Brussels, Belgium;; 5 Centre for Oncological Research, University of Antwerp, Wilrijk, Belgium;; 6 Department of Radiology, Universitair Ziekenhuis Brussel, Brussels, Belgium;; 7 Department of Pathology, Universitair Ziekenhuis Brussel, Brussels, Belgium;; 8 Experimental Pathology, Vrije Universiteit Brussel, Brussels, Belgium;; 9 mProbe, Rockville, Maryland; and; 10 Department of Nuclear Medicine, Algemeen Ziekenhuis Sint-Jan Brugge Oostende, Bruges, Belgium

**Keywords:** HER2, sdAb, PET/CT, phase II, breast carcinoma

## Abstract

Human epidermal growth factor receptor 2 (HER2) status is used for decision-making in breast carcinoma treatment. The status is obtained through immunohistochemistry or in situ hybridization. These two methods have the disadvantage of necessitating tissue sampling, which is prone to error due to tumor heterogeneity or interobserver variability. Whole-body imaging might be a solution to map HER2 expression throughout the body. **Methods:** Twenty patients with locally advanced or metastatic breast carcinoma (5 HER2-positive and 15 HER2-negative patients) were included in this phase II trial to assess the repeatability of uptake quantification and the extended safety of the [^68^Ga]Ga-NOTA-anti-HER2 single-domain antibody (sdAb). The tracer was injected, followed by a PET/CT scan at 90 min. Within 8 d, the procedure was repeated. Blood samples were taken for antidrug antibody (ADA) assessment and liquid biopsies. On available tissues, immunohistochemistry, in situ hybridization, and mass spectrometry were performed to determine the correlation of HER2 status with uptake values measured on PET. If relevant preexisting [^18^F]FDG PET/CT images were available (performed as standard of care), a comparison was made. **Results:** With a repeatability coefficient of 21.8%, this imaging technique was repeatable. No clear correlation between PET/CT uptake values and pathology could be established, as even patients with low levels of HER2 expression showed moderate to high uptake. Comparison with [^18^F]FDG PET/CT in 16 patients demonstrated that in 7 patients, [^68^Ga]Ga-NOTA-anti-HER2 shows interlesional heterogeneity within the same patient, and [^18^F]FDG uptake did not show the same heterogeneous uptake in all patients. In some patients, the extent of disease was clearer with the [^68^Ga]Ga-NOTA-anti-HER2-sdAb. Sixteen adverse events were reported but all without a clear relationship to the tracer. Three patients with preexisting ADAs did not show adverse reactions. No new ADAs developed. **Conclusion:** [^68^Ga]Ga-NOTA-anti-HER2-sdAb PET/CT imaging shows similar repeatability to [^18^F]FDG. It is safe for clinical use. There is tracer uptake in cancer lesions, even in patients previously determined to be HER2-low or -negative. The tracer shows potential in the assessment of interlesional heterogeneity of HER2 expression. In a subset of patients, [^68^Ga]Ga-NOTA-anti-HER2-sdAb uptake was seen in lesions with no or low [^18^F]FDG uptake. These findings support further clinical development of [^68^Ga]Ga-NOTA-anti-HER2-sdAb as a PET/CT tracer in breast cancer patients.

## Visual Abstract

**Figure:**
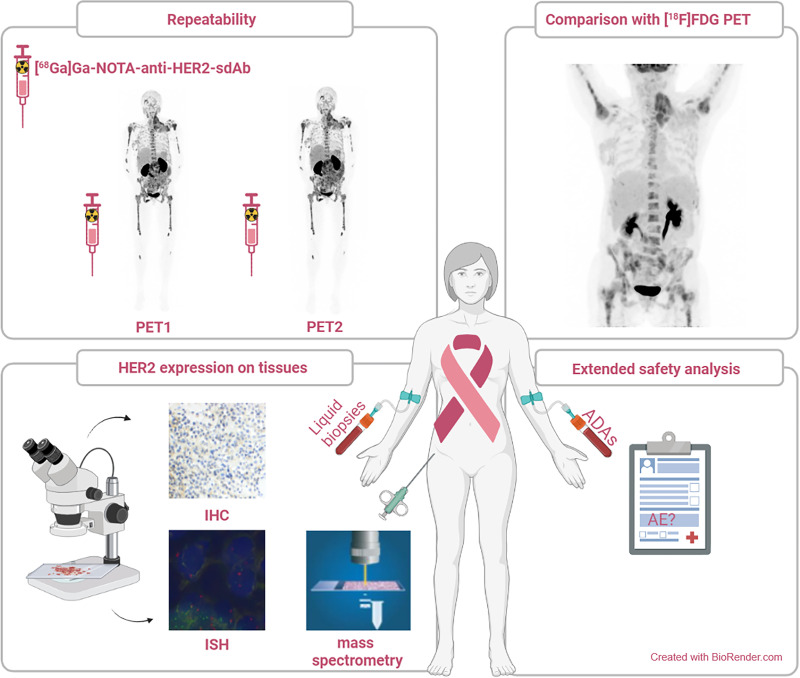

Worldwide, the most common cancer in women is breast cancer (1). The expression of both hormone receptors and human epidermal growth factor receptor 2 (HER2) is important for prognosis and therapy choices (2). Therapies targeting HER2 have been developed over the last 2 decades, including kinase inhibitors, trastuzumab, pertuzumab, and trastuzumab-based antibody–drug conjugates (3). Receptor expression and genome status are predictive biomarkers for these therapies; in HER2-positive breast carcinoma patients, these therapies improve the overall survival (4–6). Following the guidelines of the American Society of Clinical Oncology and College of American Pathologists, HER2-positive patients were defined as those with an immunohistochemistry score of 3+ or those with an immunohistochemistry score of 2+ but a positive score on in situ hybridization (ISH). In recent years, a new category emerged, termed *HER2-low* (1+ and 2+ with negative ISH), as these patients also benefit from the second-generation antibody–drug conjugates trastuzumab–deruxtecan (7). This makes the correct assessment of HER2 expression levels and the identification of such HER2-low disease even more important, as the detection of low levels of HER2 expression could be key for selecting the right drug for the right patient (5*,*^7^).

HER2 status is determined by immunohistochemistry or ISH on biopsy specimens (8). Biopsies are invasive but allow the assessment of many required molecular parameters, albeit in only a small fragment of the disease present in the patient. With increasing data confirming the heterogeneous expression of HER2 within and between cancer lesions, whole-body assessment may circumvent the need for multiple biopsies (5*,*^9^*,*^10^).

Several targeting molecules can be used as imaging tracers: monoclonal antibodies and their fragments, peptides, protein scaffolds, and others (11–13). Each has its own specific pharmacokinetic characteristics, which influences the choice of radionuclide (14). A single-domain antibody (sdAb) targeting HER2 (2Rs15D) has been developed (15*,*^16^). The sdAbs are small (12–15 kDa); they have high stability, a nanomolar affinity, and low immunogenicity (17*,*^18^). Radiolabeled sdAbs target their antigen rapidly and are eliminated by the kidneys. This implies that they can be labeled with short-lived radionuclides such as ^68^Ga or ^18^F, reducing the radioactive dose to the patient in comparison with the longer-lived radionuclides needed for radiolabeling of monoclonal antibodies. Therefore, these sdAbs allow for same-day imaging, making them ideal probes for molecular imaging.

A phase I study confirmed that [^68^Ga]Ga-NOTA-anti-HER2-sdAb PET/CT is a safe procedure with a radiation dose similar to that of other short-lived PET/CT tracers. The tracer accumulates in HER2-positive metastases, with optimal image quality at 90 min after injection (15).

The phase II trial reported here aimed to evaluate the repeatability of [^68^Ga]Ga-NOTA-anti-HER2-sdAb uptake in breast carcinoma patients (19). Additionally, tracer uptake was correlated with HER2 expression in tissues, if available, and safety and immunogenicity data for the investigational medicine were evaluated beyond phase I (17*,*^20^). Furthermore, if [^18^F]FDG PET/CT images were available, a retrospective comparison was made.

## MATERIALS AND METHODS

Complete materials and methods can be found in the supplemental material (available at http://jnm.snmjournals.org) (15*,*^21^–^23^). The protocol of this prospective open-label phase II study (EudraCT 2016-002164-13) was approved by the Belgian Federal Agency for Medicines and Health Products, the local ethics committee, and the Federal Agency for Nuclear Control. The study was performed in accordance with the declaration of Helsinki and the International Conference on Harmonization Guidelines for Good Clinical Practice. Informed consent was obtained from all subjects at inclusion. Twenty women with locally advanced or metastatic breast carcinoma (5 HER2-positive and 15 HER2-negative patients) were included between April 15, 2019, and January 12, 2021.

To assess the repeatability of this technique, patients were injected intravenously twice with [^68^Ga]Ga-NOTA-anti-HER2-sdAb with a maximal interval of 8 d and a minimal interval of 18 h, with each injection followed by a PET/CT scan after 90 min. The SUV_peak_ in selected tumor lesions (minimum diameter, 12 mm; maximum of 2 lesions per organ system and 5 per patient) and SUV_mean_ in the liver and in the left ventricle were assessed after both scans by a central reviewer. Bland–Altmann plots and calculations of the repeatability coefficient and the mean absolute percentage difference (Supplemental Table 1) were performed as described by Lodge et al. (19).

To correlate uptake values and HER2 expression within lesions, patients were given the option of undergoing a biopsy, the location of which was chosen on the basis of the SUV and feasibility. In patients who declined biopsy, other relevant tissues that were obtained as the standard of care were used if available. Immunohistochemistry analysis and ISH on the tissues were assessed in-house and reassessed elsewhere (Anapath Services). Mass spectrometry for HER2 quantification was performed by mProbe (24). Blood samples were taken to assess circulating free DNA and RNA for the presence of the HER2 gene or its transcript.

For the exploratory endpoint of interlesion heterogeneity in tracer uptake, the number of patients in the first PET/CT scan with differences in SUV_peak_ above an arbitrary threshold of 3 g/mL is reported. As no comparison at the histologic level was available, a comparison with [^18^F]FDG uptake was made in a subset of patients to evaluate if the observed difference could be purely related to partial-volume effects. Patients in whom the interlesional difference of 3 g/mL was not in line with at least a similar 50% difference in [^18^F]FDG were considered to have a potential interlesional heterogeneity.

[^18^F]FDG PET/CT, performed as the standard of care within 8 wk from [^68^Ga]Ga-NOTA-anti-HER2-sdAb PET/CT, was available for 16 patients and used for comparison.

Adverse events (AEs) were documented, and blood samples were collected before each scan and 60–365 d after the last scan to determine the presence of antidrug antibodies (ADAs). To exclude false-positive ADA results induced by the presence of the HER2 protein in the serum sample, the latter was evaluated.

## RESULTS

### Patient Characteristics

Twenty adult women (mean age, 58.6 y) were included (5 HER2-positive and 15 HER2-negative patients). Table 1 summarizes the patient characteristics.

**TABLE 1.** Patient Characteristics and Lesional Uptake Values

Patient	Age (y)	HER2 status at inclusion (IHC)	Type	ER/PR at inclusion	Optional biopsy	Mean SUV_peak_ (PET1/PET2)	Lowest SUV_peak _(PET1/PET2)	Highest SUV_peak_ (PET1/PET2)
1	62	p (3+)	Ductal	n/n	No	2.7/2.4	1.7/1.6*	3.7/3.2*
2	81	n (0)	Ductal	p/p	No	5.4/4.9	2.2/2.3^†^	11.6/9.9^‡^
3	50	p (3+)	Ductal	p/p	No	6.6/6.1	5.2/4.5^§^	8.0/7.6^ǁ^
4	70	n (1+)	Ductal	p/n	Yes	8.3/8.4	6.4/6.2^¶^	10.2/10.6^¶^
5	72	n (1+)	Ductal	p/p	No	6.4/7.0	5.6^#^	7.2^#^
6	63	n (0)	Lobular	p/p	Yes	5.3/5.6	2.9/3.4^†^	7.6/7.7^‡^
7	75	n (0)	Lobular	p/p	No	9.2*	—	—
8	37	n (2+)	Ductal	p/p	No	7.3/7.4	5.4/5.3*	10.7/11.4^¶^
9	53	n (1+)	Lobular	p/p	Yes	5.1/5.3	2.4/2.4^¶^	7.8/8.2^¶^
10	73	n (2+)	Ductal	p/p	No	1.8/1.8	1.6/1.5*	1.8/2.0^¶^
11	58	n (1+)	Ductal	n/n	No	2.6/2.5	2.4/2.2^†^	2.7/2.6^#^
12	62	n (1+)	Ductal	p/p	No	8.3/7.7	8.0/7.5^¶^	8.6/7.8^¶^
13	33	p (3+)	Ductal	p/n	Yes	—	—	—
14	65	n (2+)	Ductal	p/p	Yes	12.9/11.6	7.7/7.2^¶^	18.0/16.0^¶^
15	51	n (1+)	Ductal	p/p	No	3.5/3.3	3.1/2.7^¶^	3.9/4.0^¶^
16	46	n (1+)	Ductal	p/p	Yes	4.3^¶^	—	—
17	56	p (3+)	Ductal	n/n	Yes	—	—	—
18	57	p (3+)	Ductal	n/n	Yes	4.4^‡^	—	—
19	62	n (2+)	Ductal	n/n	No	5.3/5.8	3.5/3.8^ǁ^	8.8/9.3^‡^
20	46	n (2+)	Ductal	p/n	No	2.0/1.7	1.6/1.3^#^	2.4/2.2^#^

* Lung.

† Primary breast.

‡ Lymph node.

§ Thoracic wall.

ǁ Breast (not primary lesion).

¶ Bone.

# Liver.

IHC = immunohistochemistry; ER = estrogen receptor; PR = progesterone receptor; PET1 = first PET/CT scan; PET2 = second PET/CT scan; p = positive; n = negative.

In patients in whom only 1 lesion was assessed, values of lesions are described. Data for lesions of patient 13 and patient 17 are not included as relevant lesion was nonmalignant.

Patients were injected intravenously with 121.3 ± 15.7 MBq (50.2 ± 13.9 μg) of [^68^Ga]Ga-NOTA-anti-HER2-sdAb (range, 98.3–153.0 MBq) for the first PET/CT scan and 126.1 ± 16.9 MBq (45.3 ± 7.6 μg) of [^68^Ga]Ga-NOTA-anti-HER2-sdAb (range, 104.3–166.9 MBq) for the second PET/CT scan. Three of 20 patients were scanned only once because of withdrawal of consent (*n* = 2) or because of the severe acute respiratory syndrome coronavirus 2 pandemic (*n* = 1). In 18 of 20 patients, active metastatic disease was confirmed, either by biopsy or, in the absence of biopsy, by patient follow-up using medical imaging data or clinical examination in the case of skin metastases. In patients 13 and 17, the suggestive lesions were finally diagnosed as a relapse of tuberculosis (Supplemental Fig. 1) and lung atelectasis (Supplemental Fig. 2), respectively, rather than breast carcinoma.

### Tracer Biodistribution and Uptake in Normal Tissues

**FIGURE 1.**
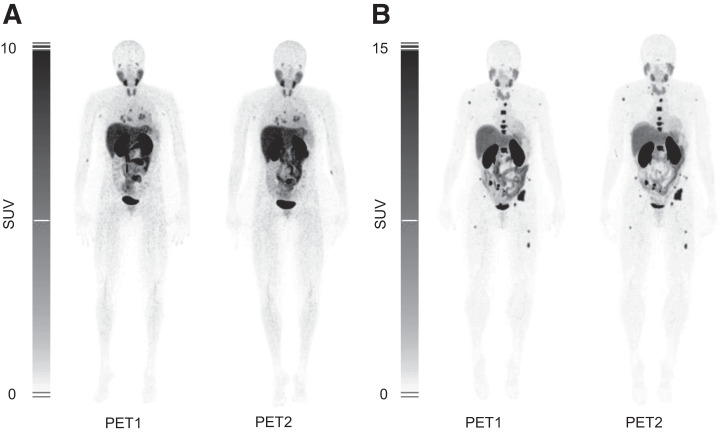
Patient 8 (A) and patient 14 (B) are both HER2-negative patients. In both patients, similar physiologic uptake in liver, kidneys, intestines, bladder, and salivary glands can be seen. Not only physiologic biodistribution but also uptake in lesions is similar between first PET/CT scan (PET1) and second PET/CT scan (PET2). In patient 8 (A), tracer uptake in lung metastases and metastases in mediastinal lymph nodes is visible. In patient 14 (B), tracer uptake in diffuse bone metastases is present.

Figure 1 shows 2 HER2-negative patients who were injected twice with [^68^Ga]Ga-NOTA-anti-HER2-sdAb. The main uptake in the kidneys, liver, and intestines was in line with the results of the phase I trial (15). For all 20 patients, the SUV_mean_ in healthy liver tissue was 11.7 ± 4.6 g/mL (range, 2.6–19.9 g/mL). In the left ventricle, with an approximation of blood-pool activity, the SUV_mean_ was 1.4 ± 0.2 g/mL (range, 0.9–2.0 g/mL).

### Tracer Uptake in Tumor Lesions and Repeatability Assessment

For each patient with cancer lesions (*n* = 18), the mean SUV_peak_ for selected lesions, as well as the maximal and minimal SUV_peak_ among the lesions, is presented in Table 2. Tracer uptake in cancer lesions ranged from an SUV_peak_ of 1.5 g/mL, which is close to blood-pool activity, to an SUV_peak_ of 18.0 g/mL. Uptake could be evaluated in 5 liver lesions in 3 patients, all with HER2-negative disease. Here, lesional uptake tended to be lower than in the surrounding liver tissue with an SUV_peak_ of 3.9 ± 2.4 g/mL (range, 1.6–7.2 g/mL), as measured on the first PET/CT scan.

**TABLE 2.** Correlation Between Tissue and Images for Individual Lesions with Contemporary Biopsy

Patient	HER2 status at inclusion	IHC/ISH at inclusion	IHC (UZB/APS)	ISH (UZB/APS)	SUV_peak_ (PET1/PET2)
2	n	0/*	0/0	—/p	2.2/2.3
			0/0	—/n	11.6/9.9
4	n	1+/*	2+/1+	p/p	6.4/6.2
5	n	1+/n	1+/1+	n/p	7.2/7.0
6	n	0/*	0/2+	n/—	7.6/7.7
7	n	0/*	0/0	n/n	9.2/^†^
8	n	2+/n	2+/3+	n/n	10.7/11.4
9	n	1+/*	1+/—	n/n	7.8/8.2
10	n	2+/n	2+/—	n/p	1.4/1.4
11	n	1+/*	0/—	—/—	2.7/2.6
		1+/*	1+/—	—/—	2.4/2.2
12	n	1+/*	1+/—	—/—	8.6/7.8
14	n	2+/n	2+/—	—/—	18.0/16.0
15	n	1+/n	0/—	—/—	3.9/4.0
16	n	1+/*	2+/2+	—/—	5.7/^†^
18	p	3+/*	3+/—	p/—	4.4/^†^
19	n	2+/n	2+/—	n/—	3.5/3.8

* Not determined in clinical routine.

† No second PET/CT scan was performed.

IHC = immunohistochemistry; UZB = Universitair Ziekenhuis Brussel; APS = Anapath Services; n = negative; p = positive; — = data not available; PET1 = first PET/CT scan; PET2 = second PET/CT scan.

**FIGURE 2.**
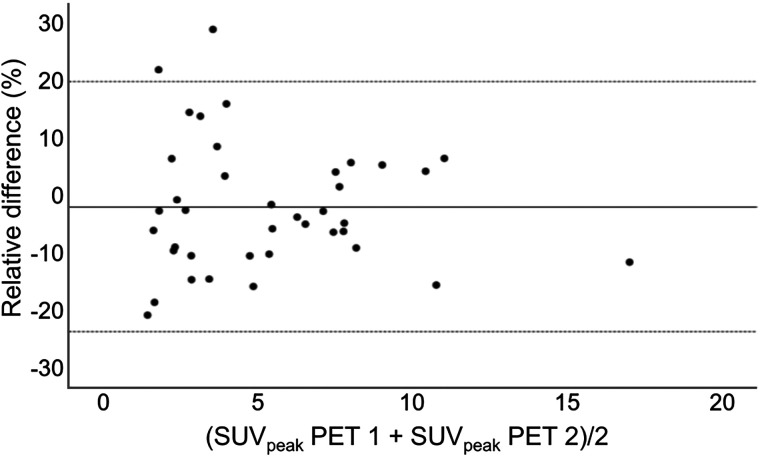
Bland–Altman plot showing SUV_peak_ difference data in relative units as function of mean. Gray dotted lines show 95% CI. PET 1 = first PET/CT scan; PET 2 = second PET/CT scan.

In total, 39 lesions in 16 patients were included in the repeatability analysis. The mean time interval between scans was 4.2 ± 1.4 d (range, 1–7 d). Lesions were located in the breasts (*n* = 8), lungs (*n* = 6), bones (*n* = 14), liver (*n* = 4), lymph nodes (*n* = 5), or elsewhere (*n* = 2). Figure 1 shows that the images were comparable visually. The distribution of the relative differences was normal, which allowed analysis via the Bland–Altman plot shown in Figure 2. The repeatability coefficient of the SUV_peak_ was 21.8%, and the mean absolute percentage difference was 9.2%.

### HER2 Assessment on Tissue Samples and Liquid Biopsies

Table 2 provides an overview of histopathologic data, from both previously obtained and contemporary biopsies, and allows head-to-head comparison with tracer uptake values of [^68^Ga]Ga-NOTA-anti-HER2-sdAb in lesions with contemporary biopsy results. Eight patients consented to a study-specific but optional biopsy. Eight patients had biopsies performed as the standard of care within 4 wk from the first PET/CT scan, enabling a relevant comparison in a total of 16 patients.

Remarkably, 2 of 15 patients with HER2-negative disease, in whom a biopsy of a lesion with high tracer uptake was performed, were recategorized as HER2-positive on the basis of a positive HER2 ISH score in combination with an immunohistochemistry result of at least 2+ (patients 4 and 10). In 3 patients with the highest lesional SUV_peak_ (>10 g/mL), 2 had an immunohistochemical score of 2+, but with a negative ISH result, therefore categorizing them as HER2-negative, whereas the remaining patient was HER2-positive. On the other hand, in lesions with an SUV_peak_ below 10 g/mL, immunohistochemistry scores ranged from 0 to 3+, without any correlation between SUV_peak_ and immunohistochemistry results (Table 2). Because of insufficient tissue, few mass spectrometry data were available (Supplemental Table 2). Assessment of circulating free DNA (Supplemental Table 3) for the presence of the HER2 gene could not confirm any positive status.

### Heterogeneity

**FIGURE 3.**
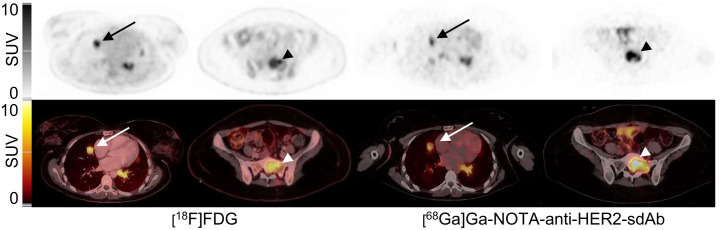
Patient 8 had bone (arrowhead), liver, and lung (arrow) metastases. SUV_peak_ with [^18^F]FDG in bone lesion (arrowhead) and in lung lesion (arrow) was 7.8 and 7.9, respectively. With [^68^Ga]Ga-NOTA-anti-HER2-sdAb, SUV_peak_ was 10.7 and 5.6, respectively, indicating difference in uptake that was not seen with [^18^F]FDG.

In 16 patients, more than 1 lesion was assessed for tracer uptake, and these data were used to study the secondary study objective of interlesional heterogeneity in tracer uptake. In 7 of these patients, the difference in the SUV_peak_ between lesions was larger than 3 g/mL (Table 1; Supplemental Table 4). Patient 8 and patient 4 are presented in Figure 3 and in Supplemental Figure 3, respectively.

### Comparison with [^18^F]FDG

**FIGURE 4.**
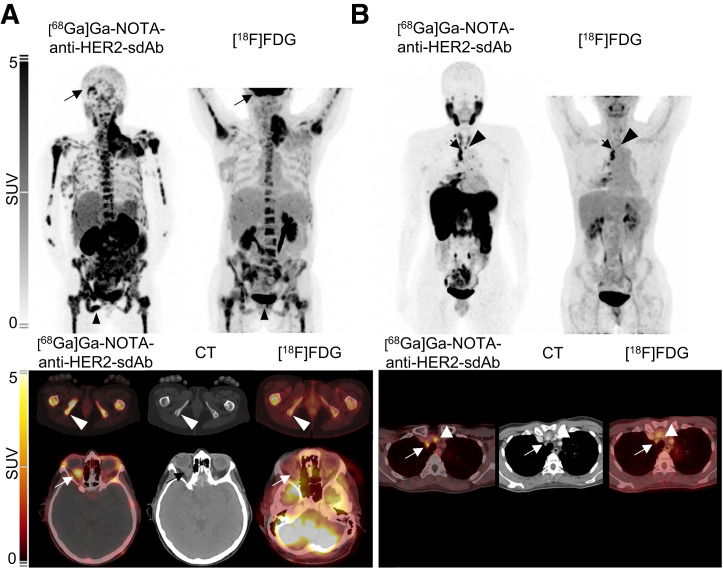
For patient 6 (A) and patient 3 (B), [^18^F]FDG (top right) and [^68^Ga]Ga-NOTA-anti-HER2-sdAb (top left) maximum-intensity projection PET images and additional axial CT (bottom center) and PET/CT (bottom left and right) images. Patient 6 was HER2-negative patient with bone metastases, with mass in neck and recent right exophthalmia. In [^68^Ga]Ga-NOTA-anti-HER2-sdAb image, uptake in these regions was confirmed, but metastatic lesions in os ischium (arrowhead) and right orbita (arrow) were better identified than on [^18^F]FDG. Patient 3 was HER2-positive patient with [^18^F]FDG-avid lymph nodes in mediastinum. Extent of disease to cervical lymph node (arrow) was better delineated on [^68^Ga]Ga-NOTA-anti-HER2-sdAb PET.

In 16 patients, [^18^F]FDG PET/CT, performed within 8 wk of the first PET/CT scan, was available for comparison. In 8 patients, lesional uptake with [^68^Ga]Ga-NOTA-anti-HER2-sdAb PET/CT could be seen in CT-graphic lesions that were not [^18^F]FDG-avid: in patients 2, 3, 12, and 19 (Supplemental Fig. 4), the [^68^Ga]Ga-NOTA-anti-HER2-sdAb PET/CT image showed additional adenopathies; in patients 4, 6, 7, and 14, additional bone metastases were revealed (Fig. 4). Two patients with suggestive [^18^F]FDG uptake in ultimately noncancerous lesions did not show HER2 tracer uptake above the blood-pool activity in these lesions (patient 14, inflammation around breast prosthesis; patient 13, tuberculosis) (Supplemental Fig. 1).

### Extended Safety Assessment

Sixteen AEs were documented and are described in Supplemental Table 5. Six AEs were reported as possibly related to [^68^Ga]Ga-NOTA-anti-HER2-sdAb; they were of mild intensity, and the patients recovered spontaneously. In retrospect, no clear relationship between these AEs and [^68^Ga]Ga-NOTA-anti-HER2-sdAb could be determined.

ADAs were detected in the serum of 3 patients, with titers ranging from 1:2 (patient 9) and 1:8 (patient 2) to 1:512 (patient 1). All 3 patients had preexisting ADAs, but no rise in the titers was observed after tracer injection. Patient 1, a HER2-positive patient, had unexpectedly low tracer uptake in metastatic lesions, but contemporary tissue analysis was not able to assess if this could be due to the loss of HER2 expression over the course of the disease or the presence of preexisting ADAs.

In patients with preexisting ADAs, the presence of circulating HER2 protein in the blood was excluded as a possible cause of false positivity. No related AEs were documented in these patients.

None of the patients without ADAs before injection developed ADAs afterward.

## DISCUSSION

The classification of patients according to HER2 status is based on immunohistochemistry analysis and ISH results. The problem of accurate HER2-status assessment, however, is compounded by the dynamics of receptor expression during the disease course and by expression heterogeneity (25). This may lead to sampling errors. Even with a current standard ERBB2 immunohistochemistry assay and following the American Society of Clinical Oncology and College of American Pathologists score, which allows different expression levels to be distinguished, the scoring accuracy in the low range (0 vs. 1+) is poor (26*,*^27^). These inaccuracies might entail giving a treatment with possible AEs to patients who will not benefit or denying patients a potentially efficacious treatment. That is why other techniques to assess the HER2 status are currently being developed using compounds ranging from mass spectrometry to molecular imaging (28–30).

The present prospective phase II study on [^68^Ga]Ga-NOTA-anti-HER2-sdAb PET/CT confirmed the overall biodistribution previously observed in the phase I trial (15), that is, normal uptake in the kidneys, liver, and intestines (16).

Evaluation of the repeatability of the SUV_peak_ in tumor lesions in 16 patients resulted in a repeatability coefficient of 22.8%. This is in line with repeatability coefficients reported for [^18^F]FDG SUV_peak_, ranging between 16% and 37%, as well as with those reported for ^68^Ga-PSMA-HBED-CC SUV_peak_ (repeatability coefficient, 33%–38%) or for [^68^Ga]GaABY-025 (a HER2-targeting Affibody [Affibody AB]) SUV_max_ (repeatability coefficient, 18%) (19*,*^31^*,*^32^). Our data thus demonstrate a high repeatability of [^68^Ga]Ga-NOTA-anti-HER2-sdAb, justifying its future quantitative use in patients.

Differences between local and central immunohistochemistry results were observed (patients 4, 6, and 8 in Table 2), in keeping with the reported lower accuracy of immunohistochemistry results in the lower range (26).

Overall, tracer uptake above background is seen in many patients with classic HER2-negative disease, mainly in HER2-low disease but also in some cases of HER2-null disease. We hypothesize that tracer uptake in HER2-null disease might reflect cases of HER2-ultralow disease that could be detected by this sensitive imaging tracer (33). However, it remains to be investigated in further clinical trials whether [^68^Ga]Ga-NOTA-anti-HER2-sdAb PET/CT imaging could outperform immunohistochemistry and ISH analyses as a method to predict treatment outcome in HER2-low breast cancer patients.

Differences were noted between the immunohistochemistry results at inclusion and at concurrent biopsies, regardless of HER2 status (Table 2). In patient 4 (HER2-negative disease at inclusion), the study-specific biopsy, performed in a lesion with high tracer uptake, yielded an immunohistochemistry score of 2+, with HER2 amplification assessed on ISH, recategorizing this patient as HER2-positive and resulting in the subsequent change to HER2-targeted treatment. This image-guided biopsy therefore had a therapeutic impact in this patient.

In 7 of 16 patients, differences in SUVs of more than 3 g/mL were found between lesions, which points at interlesional heterogeneity in tracer uptake within the same patient. It remains to be further investigated if this also correlates with heterogeneity in HER2 expression. Nevertheless, these findings already suggest a possible role for [^68^Ga]Ga-NOTA-anti-HER2-sdAb PET/CT to assess heterogeneity and to follow the dynamics of HER2 expression in the course of the disease. Tumor heterogeneity has been suggested as an explanation for the development of resistance to anti-HER2 therapies in breast carcinoma patients and might be the cause of a so-called mixed response to HER2-targeted therapy (34).

The imaging data of 16 patients were compared with routinely obtained [^18^F]FDG PET/CT images. In 3 of these patients, the earlier reported differences in [^68^Ga]Ga-NOTA-anti-HER2-sdAb uptake, which point at interlesional heterogeneity, were not found in [^18^F]FDG uptake of those same lesions, confirming that such differences are more likely due to heterogeneous expression of HER2 rather than other effects such as partial volume. In 2 patients, no uptake of [^68^Ga]Ga-NOTA-anti-HER2-sdAb PET/CT was found in lesions that took up [^18^F]FDG, but these lesions were finally confirmed to be inflammatory lesions, suggesting a higher specificity for [^68^Ga]Ga-NOTA-anti-HER2-sdAb than for [^18^F]FDG. In addition, [^68^Ga]Ga-NOTA-anti-HER2-sdAb showed improved detection of cancer lesions in 8 patients. Although [^18^F]FDG has a good detection potential in highly proliferative breast tumors, such as triple-negative and HER2-positive tumors, it might not be as sensitive in tumors with low proliferation and low metabolic activity. As [^68^Ga]Ga-NOTA-anti-HER2-sdAb uptake is seen even in lesions with a low immunohistochemistry score, this tracer might help to assess the disease extent in breast carcinoma with lower [^18^F]FDG avidity.

In comparison with [^18^F]FDG, [^68^Ga]Ga-NOTA-anti-HER2-sdAb showed uptake in lesions that were not found in 9 of 16 patients with [^18^F]FDG, suggesting a complementary value of both tracers, of which the clinical value remains to be determined in future trials. Other tracers relevant for breast carcinoma have been investigated for their ability to assess the extent of disease in breast carcinoma patients as well. [^68^Ga]GaABY-025, a HER2-targeting Affibody tracer, has been reported to be superior to [^18^F]FDG in a murine model (35).

In general, sdAbs are suggested to have a low immunogenicity risk profile (36). The extended safety assessment could not identify patients who developed ADAs after tracer injection and confirmed the low incidence of low-level preexisting ADAs without clinical safety concerns, as was also concluded by Ackaert et al. on the basis of the phase I study data of this compound (17).

Other small proteins targeting the HER2 that have advanced into clinical trials include Affibody molecules. Their biodistribution is similar to that of the sdAbs described here. [^68^Ga]GaABY-025 PET/CT had a more dichotomous distribution of SUVs in the first trial, because it could determine a threshold to distinguish HER2-positive from HER2-negative lesions (32). However, this threshold was not confirmed in a more recent trial, and their results showed a lack of correlation of [^68^Ga]GaABY-025 with immunohistochemistry data, similar to our current findings. [^68^Ga]GaABY-025 uptake did, however, predict metabolic response to HER2-targeted therapy in patients with metastatic breast carcinoma with 56% sensitivity and 66% specificity (37). [^89^Zr]Zr-trastuzumab PET/CT is able to distinguish HER2-positive from HER2-negative disease but suffers from the tracer’s long biologic half-life, necessitating scanning after several days (38).

The clinical potential of [^68^Ga]Ga-NOTA-anti-HER2-sdAb in other HER2-expressing tumor types is currently being assessed in another cohort of this phase II trial (EudraCT 2016-002164-13). An advantage of [^68^Ga]Ga-NOTA-anti-HER2-sdAb PET/CT might be its high sensitivity, with the potential to better discriminate the level of HER2 expression in a lower range than immunohistochemistry.

## CONCLUSION

[^68^Ga]Ga-NOTA-anti-HER2-sdAb PET/CT shows similar repeatability to that of [^18^F]FDG. It is safe for clinical use. It can detect HER2-expressing lesions, even in patients previously determined to be HER2-low or -negative. The tracer shows potential in the assessment of interlesional heterogeneity of HER2 expression. [^68^Ga]Ga-NOTA-anti-HER2-sdAb showed more sensitive and more specific determination of disease extent than did [^18^F]FDG in a subset of the investigated patients. Together with the ability to acquire images 90 min after injection, these findings support further clinical development of [^68^Ga]Ga-NOTA-anti-HER2-sdAb as a PET/CT tracer in breast cancer patients.

## DISCLOSURE

This project was funded by Kom op tegen Kanker (Stand Up to Cancer), the Flemish Cancer Society, and by IWT TBM (IWT150198). Odrade Gondry received an Emmanuel Van der Schueren grant funded by Kom op tegen Kanker (Stand Up to Cancer), the Flemish Cancer Society. Nick Devoogdt and Tony Lahoutte are shareholders and consultants for Precirix. Nick Devoogdt, Tony Lahoutte, and Marleen Keyaerts are shareholders and consultants for Abscint NV/SA, which has licensed a patent on the diagnostic HER2-sdAb tracer described in this study. Sheeno Thyparambil works for mProbe. Catarina Xavier, Nick Devoogdt, Tony Lahoutte, and Marleen Keyaerts hold patents related to sdAb imaging and therapy. Tony Lahoutte and Marleen Keyaerts have an FWO clinical mandate. No other potential conflict of interest relevant to this article was reported.

## Notes

KEY POINTS **QUESTION:** The aim of this trial is to assess the repeatability, the safety, and the clinical potential of [^68^Ga]Ga-NOTA-anti-HER2-sdAb PET/CT as an imaging technique in breast carcinoma. **PERTINENT FINDINGS:** This phase II clinical trial indicates that the technique is safe and repeatable and warrants further investigation into its clinical potential, especially in HER2-low patients. **IMPLICATIONS FOR PATIENT CARE:** This technique allows us to assess the presence of HER2 overexpression in a sensitive way throughout the whole body, helping to identify patients eligible for new HER2-targeting therapies.
